# NGS analysis of collagen type I genes in Polish patients with Osteogenesis imperfecta: a nationwide multicenter study

**DOI:** 10.3389/fendo.2023.1149982

**Published:** 2023-09-22

**Authors:** Kinga Sałacińska, Iwona Pinkier, Lena Rutkowska, Danuta Chlebna-Sokół, Elżbieta Jakubowska-Pietkiewicz, Izabela Michałus, Łukasz Kępczyński, Dominik Salachna, Nina Wieczorek-Cichecka, Małgorzata Piotrowicz, Tatiana Chilarska, Aleksander Jamsheer, Paweł Matusik, Małgorzata Wilk, Elżbieta Petriczko, Maria Giżewska, Iwona Stecewicz, Mieczysław Walczak, Magda Rybak-Krzyszkowska, Andrzej Lewiński, Agnieszka Gach

**Affiliations:** ^1^ Department of Genetics, Polish Mother’s Memorial Hospital Research Institute, Lodz, Poland; ^2^ Department of Bone Metabolic Diseases, University Centre of Paediatric, Medical University of Lodz, Lodz, Poland; ^3^ Department of Pediatrics, Newborn Pathology and Bone Metabolic Diseases, Medical University of Lodz, Lodz, Poland; ^4^ Department of Endocrinology and Metabolic Diseases, Polish Mother’s Memorial Hospital Research Institute, Lodz, Poland; ^5^ Department of Medical Genetics, Poznan University of Medical Sciences, Poznan, Poland; ^6^ Department of Pediatrics, Pediatric Obesity and Metabolic Bone Diseases, Faculty of Medical Sciences in Katowice, Medical University of Silesia, Katowice, Poland; ^7^ Department of Pediatrics, Endocrinology, Diabetology, Metabolic Disorders and Cardiology of Developmental Age, Pomeranian Medical University, Szczecin, Poland; ^8^ Department of Obstetrics and Perinatology, University Hospital, Kraków, Poland

**Keywords:** osteogenesis imperfecta, connective tissue disorder, collagen type I, COL1A1, COL1A2, next-generation sequencing

## Abstract

Osteogenesis imperfecta (OI) is a rare genetic disorder of the connective tissue. It presents with a wide spectrum of skeletal and extraskeletal features, and ranges in severity from mild to perinatal lethal. The disease is characterized by a heterogeneous genetic background, where approximately 85%–90% of cases have dominantly inherited heterozygous pathogenic variants located in the *COL1A1* and *COL1A2* genes. This paper presents the results of the first nationwide study, performed on a large cohort of 197 Polish OI patients. Variants were identified using a next-generation sequencing (NGS) custom gene panel and multiplex ligation probe amplification (MLPA) assay. The following OI types were observed: 1 (42%), 2 (3%), 3 (35%), and 4 (20%). Collagen type I pathogenic variants were reported in 108 families. Alterations were observed in α1 and α2 in 70% and 30% of cases, respectively. The presented paper reports 97 distinct causative variants and expands the OI database with 38 novel pathogenic changes. It also enabled the identification of the first glycine-to-tryptophan substitution in the *COL1A1* gene and brought new insights into the clinical severity associated with variants localized in “lethal regions”. Our results contribute to a better understanding of the clinical and genetic aspects of OI.

## 1 Introduction

Osteogenesis imperfecta (OI) belongs to a group of rare genetic disorders with a prevalence of 1–5/10,000 (ORPHA 666). The disease is associated with aberrations of collagen type I; as this structural protein is found in various connective tissues, the condition is characterized by a wide spectrum of clinical features. The most characteristic include fractures following minor trauma, bending of long bones, and growth deficiencies. In addition, craniofacial (macrocephaly, flat midface, and triangular-shaped face), chest, and spinal deformities are observed. Patients may also develop extraskeletal features, such as blue-colored sclerae, hearing loss, abnormal dentition (dentinogenesis imperfecta), joint hyperlaxity, reduced lung function, and cardiac defects (1, 2).

According to the current classification, OI is divided into five clinical types: OI type 1—non-deforming with persistently blue sclerae; OI type 2—a perinatal lethal form; OI type 3—characterized by progressive deformation; OI type 4—a moderate form with normal sclerae; OI type 5—with calcification of the interosseous membranes and/or hypertrophic callus (3, 4). This subdivision corresponds to the severity of symptoms, based on clinical and radiological features, without taking into account the genetic background.

In approximately 85%–90% of cases, the genetic cause of OI is associated with dominantly inherited mutations in the *COL1A1* and *COL1A2* genes encoding collagen type I molecules, resulting in quantitative or qualitative aberrations (1, 4). Among patients negative for collagen type I mutations, expanded molecular testing has revealed the presence of causative variants in several non-collagen genes. According to the OMIM (Online Mendelian Inheritance in Men) classification, 22 types of the disease have been described; however, the list is constantly expanding (5). Types I–IV are associated with mutations in genes encoding collagen type I, while the remainder are linked to genes encoding proteins involved in its biosynthesis, folding, post-translational modifications, bone mineralization, and osteoblast differentiation (6–8).

Although research into OI commenced over four decades ago, several issues concerning its genetic and clinical aspects remain challenging. This paper presents the results of a population-based study performed in a group of Polish patients with OI. It is the first nationwide study to describe the genetic background of this rare disease. We hope that our findings will support a wide range of healthcare professionals during clinical diagnosis and molecular screening.

## 2 Materials and methods

### 2.1 Subjects

The study population included 197 individuals from 180 families suspected of OI recruited by six Polish medical health centers in the years 2016–2022. The study group comprised prenatal and postnatal cases, aged 3 months to 46 years at the time of assessment. Patients were of Caucasian origin and presented a variable spectrum of clinical features. Medical information was derived from a questionnaire and medical records. Clinical evaluation was performed according to the Sillence classification. Informed consent was obtained from all participants or their legal guardians.

### 2.2 DNA isolation

Genomic DNA was automatically isolated from peripheral blood leucocytes using MagCore Genomic DNA Whole Blood Kit (RBC Bioscience, Taiwan). A manual Sherlock AX kit (A&A Biotechnology, Poland) was used for the amniotic fluid and umbilical cord.

### 2.3 Next-generation sequencing

Next-generation sequencing (NGS) was used to resequence the exons and flanking non-coding sequences of the selected genes. Two slightly different custom gene panels were applied, as the production of the reagents has been discontinued in the meantime. For 166 patients, the panel included genes for OI types I–XVII, OI-related disorders, and those that may participate in the pathogenesis of OI (9). For 31 patients, the gene set covered OI types I–XIX and OI-related disorders. Both panels were designed using Illumina DesignStudio software according to the GenBank reference for GRCh37. Libraries were prepared according to the manufacturer’s protocol for TruSeq Custom Amplicon and AmpliSeq On-Demand kits (Illumina, USA). The pooled libraries were sequenced on the MiniSeq platform (Illumina, USA).

### 2.4 Data analysis

Bioinformatics data processing, read quality control, and primary variant filtering were performed using VariantStudio software (Illumina, USA). Potentially causative mutations were selected according to the internal pipeline and analyzed using the Varsome interpretation tool (https://varsome.com/). Mutations were checked for population frequency values for the European non-Finnish population (gnomAD Exomes, gnomAD Genomes), and the impact on genes or gene products was determined according to *in silico* predictive programs. Moreover, variant databases (dbSNP, ClinVar, HGMD, and LOVD) and available literature were checked for additional information. For previously undescribed variants, the conservation score was performed using Multiple Sequence Alignment analysis across species (Clustal Omega 1.2.4; Jalview 2.11.0 software). Finally, variant classification was performed according to the ACMG guidelines (1—pathogenic, 2—likely pathogenic, 3—variant of uncertain significance, 4—likely benign, 5—benign).

### 2.5 Sanger sequencing

Targeted Sanger sequencing was performed to validate the presence of candidate variants revealed by NGS [*COL1A1*: LRG_1(t1), NM_000088.4; *COL1A2*: LRG_2(t1), NM_000089.4]. The results considered variants that were pathogenic, likely pathogenic, or of unknown significance. In case of a confirmed mutation, family screening was made. PCR products were prepared with custom-designed primers, cleaned (ExoSAP-IT, Applied Biosystems, USA), and labeled (BigDye Terminator v3.1 Cycle Sequencing Kit; Applied Biosystems, USA). After sequencing on an 8-capillary 3500 Genetic Analyzer (Applied Biosystems, USA), the results were analyzed with Mutation Surveyor V5.1.0 software (SoftGenetics, USA).

### 2.6 Multiplex ligation probe amplification

Patients negative for NGS variant filtering were subjected to multiplex ligation probe amplification (MLPA) analysis of the *COL1A1* and *COL1A2* genes (SALSA MLPA P271 and P272 kit, MRC Holland).

### 2.7 Statistical analysis

Phenotypic severity was assessed using the *χ* ^2^ Cochrane-Armitage test for trend and stepwise Bonferroni correction for multiple comparisons (Bonferroni-Hochberg procedure). Values of *p* < 0.05 were treated as statistically significant. Statistical analysis was performed using The R Project for Statistical Computing software (v. 4.2.1).

## 3 Results

### 3.1 The study population

The targeted NGS analysis was performed for 197 patients (180 families), i.e., 168 simplex and 12 family cases (29 patients). After careful reconsideration, eight patients were excluded from general calculations. Of this group, five individuals had insufficient indications for examination, needed differential diagnosis, or presented an unclear phenotype, while the other three were unaffected family members, i.e., parents of siblings with severe and lethal forms (Family 11) and a 4-month-old girl with a family history of OI (Family 6).

Finally, 189 patients from 175 families presented features of OI and were included in further analysis. The study population consisted of two fetuses, 137 children and teenagers (i.e., below 18 years old) and 50 adults. Prior to genetic testing and based on available clinical information, 107 patients (96 families) were classified as having mild type 1, 34 (32 families) had moderate type 4, and 45 (44 families) were assigned to progressively deforming type 3. The study population included three cases of lethal OI type 2. The first child had been tested prenatally from the amniotic fluid at 12 weeks of gestation but died before labor; the second was stillborn and had post-mortem screening performed from an umbilical cord; the third had a molecular test performed from a blood sample at the age of 6 months, and died before the age of 1 year.

### 3.2 Genetic background

Causative variants were identified in 108 families (119 patients), in 106 cases (117 patients) using the NGS technique, and in the other two using the MLPA assay. In four probands, two potentially pathogenic changes located in the collagen type I genes were reported (*Section 3.2.4*). Finally, for each patient, only one variant suspected to have a more significant impact on clinical manifestation was included in the general calculations. Briefly, for the *COL1A1* and *COL1A2* genes, pathogenic variants were found in 76 (85 individuals) and 32 (34 individuals) families, respectively. In the study cohort, nine variants were observed in at least two unrelated families; i.e., c.1001delC, c.1678G>A, c.1821 + 1G>A, c.2596G>A, c.3076C>T, c.3118G>A and c.3421C>T were noted in *COL1A1*, while c.892G>A and c.2845G>A were found in *COL1A2*. No causative variants were found in the collagen type I genes in 67 families (70 patients) (**Table 1**).

**Table 1:** Distribution of OI types in the study population of 175 families.

OI type	Number of families with COL1A1 variant	Number of families with COL1A2 variant	Total number of families with identified variant	Number of families without identified variant	Total
**1**	40	5	45 (42%)	51	96 (55%)
**2**	3	0	3 (3%)	0	3 (1%)
**3**	25	13	38 (35%)	6	44 (26%)
**4**	8	14	22 (20%)	10	32 (18%)
**Total**	76	32	108	67	175

#### 3.2.1 Collagen type I genes variant analysis

A total of 97 distinct alterations were identified, i.e., 68 located in the *COL1A1* and 29 in the *COL1A2* gene. Of these, 38 have not been described previously (**Supplementary Materials**; **Table 1**).

Only two large rearrangements identified through the MLPA assay include a 25-exon deletion in *COL1A1* and a 6-exon deletion in *COL1A2*, which were found in patients with mild and moderate phenotypes, respectively.

Using the NGS custom gene panel, we identified 53 glycine substitutions, 14 single-nucleotide deletions, 13 splice site alterations, 10 nonsense variants, 1 in-frame deletion, and 1 in-frame insertion. In *COL1A1*, point mutations were evenly distributed along the entire gene. The vast majority were located in the triple helical region, except for three mapped to the N-terminal (c.68C>T, c.189C>A, c.386delC) and three located in the C-terminal regions (c.4171C>T, c.4332dupC, c.4248 + 1G>A). In the *COL1A2*, all variants were mapping to the triple helical domain, starting from intron 14 (**Supplementary Materials**; **Table 1**).

Frameshift and nonsense variants, identified solely in the α1 chain, were exclusively associated with mild outcomes. Splice site alterations, reported in both collagen genes, caused a range of non-lethal phenotypes. A single in-frame deletion and insertion, located in the α2 chain, caused progressively deforming and moderate outcomes, respectively. The most frequently reported missense mutations resulted in all four clinical types of OI, of which the majority lead to progressively deforming (*n* = 30) and moderate (*n* = 16) manifestations (**Table 2**).

Table 2Number and severity of variants reported in *COL1A1* and *COL1A2* genes.

**Table:** 

COL1A1					
	OI 1	OI 2	OI 3	OI 4	Total
**Frameshift**	15	–	–	–	15
**Nonsense**	10	–	–	–	10
**Splicing**	9	–	3	1	13
**Missense**	1	3	20	6	30
**Total**	35 (52%)	3 (4%)	23 (34%)	7 (10%)	68

**Table:** 

COL1A2					
	OI 1	OI 2	OI 3	OI 4	Total
**Frameshift**	–	–	–	1	1
**Splicing**	–	–	1	2	3
**In-frame deletion**	–	–	1	–	1
**In-frame insertion**	–	–	–	1	1
**Missense**	3	–	10	10	23
**Total**	3 (10%)	0 (0%)	12 (42%)	14 (48%)	29

The number and severity of particular amino acids substituting for glycine vary between collagen type I genes. Among eight different amino acids, the most prevalent were serine substitutions, with 22 distinct variants identified in the study population. Together with arginine and valine, it was associated with all clinical types of the disease. Alanine, cysteine, and aspartic acid substitutions caused moderate and progressively deforming phenotypes, while single glutamic acid and tryptophan substitutions were associated with progressively deforming OI (**Table 3**).

**Table 3:** Number and severity of amino acids substituting for glycine reported in *COL1A1* and *COL1A2* genes.

Amino acid	ALA		ARG		ASP		CYS		GLU		SER		TRP		VAL		Total	
OI Type/Gene	*COL1A1*	*COL1A2*	*COL1A1*	*COL1A2*	*COL1A1*	*COL1A2*	*COL1A1*	*COL1A2*	*COL1A1*	*COL1A2*	*COL1A1*	*COL1A2*	*COL1A1*	*COL1A2*	*COL1A1*	*COL1A2*	*COL1A1*	*COL1A2*
**OI 1**	0	0	1	0	0	0	0	0	0	0	0	2	0	0	0	1	1	3
**OI 2**	0	0	1	0	0	0	0	0	0	0	1	0	0	0	1	0	3	0
**OI 3**	5	0	1	3	0	2	2	1	1	0	9	3	1	0	1	1	20	10
**OI 4**	2	0	0	1	0	1	0	1	0	0	1	6	0	0	3	1	6	10
**Total**	7	0	3	4	0	3	2	2	1	0	11	11	1	0	5	3	30	23

#### 3.2.2 Novel variants

A total of 38 novel pathogenic variants were identified, i.e., 24 located in the *COL1A1* gene and 14 located in the *COL1A2* gene. Among these, 19 new missense changes were found, and these resulted in the full range of OI types. Single arginine and aspartic acid substitutions caused progressively deforming and moderate outcomes, respectively, while the alanine and cysteine variants resulted in both progressively deforming and moderate phenotypes. The most variable clinical manifestation was observed for serine and valine substitutions: in the α2 chain, they resulted in all non-lethal forms of the disease. Only one novel lethal variant was associated with valine substitution located in exon 19 of the α1 chain.

Among seven distinct amino acids altering conservative glycine residues, we reported the first tryptophan substitution located in the *COL1A1* gene. The novel variant c.733G>T p.(Gly245Trp), found in a patient with OI type 3, has been described in a separate publication by our team (10). A single in-frame deletion and insertion were observed in patients with progressively deforming and moderate outcomes, respectively; both were located in the α2 chain. Regardless of localization in the α1 chain, seven novel frameshift and two nonsense variants caused OI type 1. A 25-exon deletion, encompassing exons 1–25 of the *COL1A1* gene, was identified in a family with mild phenotype, while deletion of exons 6–12 of the *COL1A2* gene was associated with a moderate manifestation. The six splicing variants resulted in mild and progressively deforming phenotypes; however, no pattern was found at the DNA level that could explain its variable severity (**Supplementary Materials**; **Table 1**).

#### 3.2.3 Lethal regions

In the study population, eight patients presented nonlethal phenotypes with pathogenic glycine substitutions mapped in the “lethal regions”. Two mutations were located in the *COL1A1* (c.2984G>C and c.2993G>C) gene and five in the *COL1A2* (c.1667G>T, c.2539, c.2845G>A, c.2873G>T, and c.3215G>T) gene. Patients harboring six of these variants have been described in detail in our separate publication (9). The newly reported mutation c.2873G>T was identified in a 13-year-old boy presenting OI type 1. The patient with no extraskeletal features, a history of three vertebral fractures, and severe osteoporosis had recently started bisphosphonate treatment.

#### 3.2.4 Patients harboring two variants

In the present study, four patients were found to harbor two alterations with potential causative impact located in the collagen type I genes, i.e., variant class 1 or 2 in conjunction with variant class 2 or 3 (**Supplementary Materials**; **Table 2**).

The first patient (P7569) with OI type I harbors a known pathogenic deletion c.3008delC and a novel substitution c.992C>T p.(Ala331Val) located in the *COL1A1* gene. According to the ACMG guideline, the novel variant was classified as likely pathogenic. The performed family screening revealed the presence of both changes in the patient’s mother, who also presented an OI phenotype. Owing to the cis-allele location of the variants, the frameshift that causes NMD is probably of overriding importance and is responsible for the mild manifestation observed in the patient and his mother.

The second individual (P25) was classified with OI type III. The patient demonstrated a novel mutation c.3182G>T p.(Gly1061Val) in conjunction with a known variant c.4081G>A p.(Glu1361Lys) located in the *COL1A1* gene. The LOVD and HGMD databases have reported similar glycine substitutions in the same protein position, *viz.*, p.Gly1061Asp (CM9000070) and p.Gly1061Ser (CM940294), both resulting in OI type II. The non-glycine substitution has been described in the LOVD as pathogenic; however, no article is available. Interestingly, the Varsome interpretation tool, dbSNP, and ClinVar databases classify p.(Glu1361Lys) as a variant of unknown significance. Similarly, in the HGMD, it is linked to a publication that describes a variant in position p.1361 (CM1411904); however, this results in a glycine-to-valine substitution located in the *COL1A2* gene. As the non-glycine substitution was later identified in the patient’s unaffected mother and siblings, we suggest variant reclassification.

The third case (P55) was attributed to OI type III, which harbors glycine and non-glycine substitutions located in the *COL1A1* and *COL1A2* genes, respectively. The first variant c.2155G>A p.(Gly719Ser), of *de novo* appearance, has been previously reported as a single causative alteration in several databases in patients with OI type III. The second variant c.2123G>A p.(Arg708Gln), inherited from the unaffected mother, was classified as likely pathogenic. However, the ClinVar database and several publications indicate it to have a benign impact. Similarly to our observed case, a non-glycine change has previously been reported with a different pathogenic glycine substitution (11). According to these findings, variant c.2123G>A should be reclassified as benign.

The last patient (P103) has a known splicing alteration c.2452-2A>G located in the *COL1A1* gene and a novel non-glycine substitution c.2642A>C p.(Glu881Ala) in the *COL1A2* gene. The newly identified variant has uncertain clinical significance and has been lately described with a different splicing mutation (12). Hence, the c.2642A>C variant inherited from an unaffected mother should be recognized as benign.

### 3.3 Phenotype analysis

The prepared clinical characteristics of Polish OI patients cover only individuals with a proven molecular diagnosis of the disease. According to this, 55 patients represented mild type 1, 38 represented progressively deforming type 3, and 23 represented moderate type 4 (**Table 4**). In the study population, only three individuals had lethal OI type 2. Because of such little representation, they were not included in the statistical analysis; however, they were described at the end of the section.

**Table 4:** Clinical characteristics of the study population after molecular testing divided by OI type.

	OI type I	OI type IV	OI type III	p-Value
**Number of patients**	55	23	38	n/a
**Sex, M/F**	27/28	10/13	18/20	n/a
**Age**	4 mo–46 yo	3–37 yo	6 mo–37 yo	n/a
**Height (centile)**	<3 to 97	<3 to 25–50	<3 to 3–10	n/a
**No. of fractures**	0–38	2–40	1–130	n/a
**No. of fractures per year**	0–3.13	0.06–3.67	0.46–15.38	n/a
**Prenatal deformations/fractures**	1/54 (2%)	5/17 (29%)	35/37 (95%)	<0.05
**Vertebral fractures**	21/53 (40%)	6/23 (26%)	3/37 (8%)	<0.05
**Chest deformations**	12/54 (22%)	12/23 (52%)	30/38 (79%)	<0.05
**Upper limbs deformations**	9/54 (17%)	8/23 (35%)	34/38 (89%)	<0.05
**Lower limbs deformations**	16/54 (30%)	16/23 (70%)	38/38 (100%)	<0.05
**Mobility problems**	8/55 (16%)	9/23 (43%)	25/29 (86%)	<0.05
**Triangular shaped face**	13/53 (25%)	8/20 (40%)	24/37 (65%)	<0.05
**Dentinogenesis imperfecta**	8/55 (15%)	13/23 (59%)	25/38 (74%)	<0.05
**Blue sclerae**	50/54 (93%)	18/23 (78%)	35/38 (92%)	>0.05
**Hearing impairment**	5/55 (9%)	3/23 (14%)	5/38 (13%)	>0.05
**Osteopenia/osteoporosis**	33/45 (73%)	18/22 (82%)	21/21 (100%)	>0.05
**Pharmacological treatment**	14/55 (28%)	15/23 (75%)	35/38 (92%)	n/a
**Inheritance F/S/U**	29/6/20	7/6/10	2/18/18	n/a

mo, months old; yo, years old; F, familial; S, sporadic, U, unknown, n/a, not analyzed.

Differences in total number of patients for particular features are caused by missing data or when assessment was not applicable.

The study population demonstrated nearly equal representation of male and female patients (55 vs. 64). Regardless of the OI type, the age of the patients at the time of molecular assessment spanned from infancy to adults in their mid-40s.

The height distribution was concordant with the general phenotypic characteristics of different OI types. Patients attributed to mild type 1 covered the entire scope of the height percentile grid, while the height range significantly decreased with increasing severity of symptoms. The mean number of low-energy trauma per year also increased with disease severity, i.e., 0.65 for mild, 1.10 for moderate, and 3.25 for the progressively deforming group. However, a specific number of fractures for individual probands varied within and among OI types. In the study population, only three patients presenting mild type 1, aged 4 months (P10437), 6 months (P105), and 9 years (P835), did not experience any injury. In contrast, the highest numbers, *viz.*, 100, 120, and 130 fractures, occurred in older patients aged 14, 11, and 35 years (P33, P115, and P2208), respectively. They were all diagnosed as having OI type 3 and experienced their first fracture in the prenatal or neonatal period. Most of the probands with moderate and progressively deforming manifestations experienced their first fracture before 12 months of age. In turn, the majority of individuals with mild outcomes sustained their first fracture between 12 and 24 months of age, which falls in the period of upright standing. Interestingly, the latest first fracture occurred similarly in all groups at the age of 6 to 8 years.

The performed analysis indicates that prenatal presentation, triangular-shaped face, dentinogenesis imperfecta, vertebral fractures, chest and long bone deformations, and consequently mobility problems were significantly associated with particular OI types. For each of these features, the number of affected patients increased with disease severity. Only vertebral fractures showed a reverse trend. Interestingly, prenatal presentation, observed as *in utero* fractures or skeletal deformations, was reported in a single patient with a mild phenotype. Although the patient demonstrated bone deformations, detected by prenatal ultrasound, no fractures were sustained after birth, and only slightly bent upper and lower limbs, and blue sclerae, were noted (P10437).

Among mild cases, any reported mobility problems were mostly periodical and occurred after fractures. Walking with support was most commonly noted in the moderate group, and could be related to more severe bone deformations and valgus of the knee joints. Only one patient with type 4 was using a wheelchair due to skeletal deformities and reduction of muscle tone. In the type 3 group, 10 patients had mobility problems, 13 used a wheelchair, and 2 were immobile because of respiratory problems and delayed psychomotor development. The use of pharmacological treatment among patients with different OI types is in line with the increasing severity of skeletal deformations and the number of fractures. Treatment commencement ranged between the ages of 2 and 34 years in patients with a mild phenotype, between 1 month and 15 years for individuals with moderate outcome, and between the newborn period and 17 years in the most severe cases.

Interestingly, the presence of blue sclerae, hearing impairment, and low BMD (body mineral density) were not significantly related to the severity of OI. Blue sclerae were reported in the majority of patients enrolled in the study (90%) representing all non-lethal OI types, i.e., 93% of OI type 1 cases, 92% of OI type 3, and 78% of OI type 4. Hearing impairment was the least prevalent extraskeletal feature among OI patients, with frequency ranging between 9% and 14% of cases. Lowered BMD observed as osteopenia and osteoporosis was diagnosed in 72 out of 88 individuals, and applies to the results at the time of evaluation, regardless of the therapy. For 28 patients, the BMD value was missing; i.e., BMD was not evaluated in OI type 1 cases, perhaps due to very mild outcome, or in OI type 3, this was due to difficulty in performing densitometry tests in infants and young children.

Three cases of lethal OI were reported in the present study. A novel variant c.1247G>T p.(Gly416Val) was identified in a male fetus presenting features of OI in week 12 of gestation. The performed ultrasound examination showed bowing and shortening of long bones, chest deformations, and poor skull calcification. A second mutation c.2533G>A p.(Gly845Arg) was reported in an 11-month-old girl primarily diagnosed with very severe progressively deforming OI type 3 with intrauterine fractures, chest and long bone deformities, and respiratory problems. Eventually, she died soon after the molecular test was performed, indicating long-surviving OI type 2B (13). A third mutation c.3541G>A p.(Gly1181Ser) was observed in a male fetus with skeletal abnormalities and fractures visible at week 13 of gestation. Preterm delivery occurred at week 34 of pregnancy; however, the baby was stillborn. Variants c.2533G>A and c.3541G>A have been already noted in the lethal cases (14).

## 4 Discussion

Our analysis of 108 families in a Polish population with genetically confirmed OI found OI types 1, 2, 3, and 4 to account for 42%, 3%, 35%, and 20%, respectively. This is very close to a previous clinical study performed by Rusińska et al. (44/33/21%), as well as for the Ukrainian cohort published by Zhytnik et al. (47/17/34%) (12, 15). Interestingly, in Italian and Scandinavian populations, the percentage of mild type 1 was relatively higher (68%–77%) (16–18).

Depending on the clinical type, the applied methods allowed for molecular confirmation of the disease in 47% to 100% of tested families. A higher success rate was associated with more severe manifestations.

Overall, no pathogenic variant in the collagen type I genes was found in 38% of families. In this group, probable causative mutations were detected in non-collagen genes in 12 cases; however, family segregation or functional studies are needed to confirm the pathogenicity of the variants. We suspect that some of the undiagnosed patients presenting uncertain manifestations, with only bone fragility and no extraskeletal features, may have isolated osteoporosis. However, as the clinical outcome of OI is very broad, it cannot be excluded that the patients may have displayed an ultra-mild presentation associated with yet unknown genetic loci. In the remaining patients, we plan to expand our diagnostic methodology with WES or WGS. In other European population studies, no mutations were found in 10% to 36% of probands (12, 16–18).

The genetic background analysis of OI in the present Polish population found that 70% demonstrated the α1 pathogenic variant and 30% demonstrated the α2 variant; this result is the same as described in the literature (4). However, the percentage of qualitative (65%) and quantitative (35%) variants in collagen type I genes noted in the Polish population differed from the results in other European cohorts, where structural alterations account for 30% to 57%, respectively (12, 16–19).

Single-nucleotide variants and small deletions/duplications were detected using an NGS custom gene panel and constituted 98% of the identified changes. Large deletions and duplications, which usually account for 1%–2% of the OI cases, were identified in two individuals with a commercial MLPA assay (4).

It has been widely observed that variants causing an OI phenotype are usually unique and rarely repeated between unrelated individuals (4). In the present study cohort, no hot spot was found to be characteristic of the studied Polish OI population, as only 9 out of 97 pathogenic changes occurred, in a maximum of three unrelated families. Therefore, among the 108 families presented in this paper, 88 were identified to have a “private mutation”. Interestingly, unrelated patients harboring a common variant were found to present the same clinical type.

Our findings reveal the presence of 38 previously unreported pathogenic variants. Although the number of novel mutations is surprisingly high (39% of all identified changes), they are commonly observed in other cohorts, i.e., Swedish 31%, Ukrainian 43%, Estonian 50%, and Finnish 84% (12, 17–19).

The clinical outcome of the newly identified alterations, as well as previously reported variants, is in line with the general rule regarding the quantitative or qualitative effects of the mutation on the collagen chain (6, 7, 20, 21). According to this, all single-nucleotide deletions and nonsense variants caused OI type 1. This also includes a large 25-exon deletion containing the first exon of the *COL1A1* gene resulting in the null-allele and mild presentation. The variable clinical outcome of reported splicing alterations is also consistent with previous findings. Since several factors influence this complex process, it is not surprising that mutations altering exon–intron junctions may result in a full phenotypic range from mild to lethal (20, 22). Owing to the variable effect of splicing mutations on the collagen chains, it is hard to define how a particular variant affected mRNA without further functional studies. Thus, we suspect that nine changes lead to intron retention or exon skipping with frameshift (OI type 1 cases), while seven caused in-frame alteration (OI type 3 and 4 cases). Similarly, the six-exon deletion reported in the *COL1A2* gene most probably caused an in-frame alteration and thus OI type 4. The very rare in-frame mutations reported in the present study resulted in OI types 3 and 4. Although they are categorized as structural aberrations, the phenotype often remains unknown, as some variants can undergo NMD (4, 20).

The outcome of the identified missense variants leading to qualitative changes is mostly in line with previous findings, as they were predominantly reported in patients with severe phenotypes (i.e., 92%) (4, 8, 21).

Because of the highly variable manifestation of missense mutations, over the years, several factors have been considered to determine their severity (4, 21, 23). The heterogeneous nature of glycine substitutions was correlated with its position in the alpha chain, with greater clinical severity observed closer towards the C-terminal end due to its essential role in triple-helix formation (24–27). Based on obtained data, the gradient model appears to be valid to some extent. Thus, we are reluctant to draw any reliable conclusions based on the overall number of reported mutations (30 in α1 and 23 in α2) and particular amino acids substituting for glycine (1–11 in a single gene).

It has also been hypothesized that the phenotype of substituting residue is correlated with its identity to glycine; indeed, the majority of charged (arginine, aspartic acid, and glutamic acid) and hydrophobic (valine) amino acids result in severe outcomes (4, 6, 27). This rule also applied to the *COL1A1* tryptophan for glycine substitution c.733G>T, p.(Gly245Trp) identified in our study for the first time. As tryptophan is a large, non-polar aromatic amino acid containing an indole side chain, the observed progressively deforming phenotype is in line with this principle. In contrast, small amino acids (alanine, cysteine, and serine) correlated with a milder outcome, were mostly responsible for OI types 3 and 4. Interestingly, the mild and lethal cases reported in our study were caused by the same amino acids, i.e., small serine, charged arginine, and hydrophobic valine.

Referring to the regional model, significant mortality has been associated with mutations mapping to two clusters dispersed along *COL1A1*, and eight dispersed along *COL1A2* (2, 23, 27). Despite their crucial role in maintaining interactions between collagen and other extracellular molecules, variants altering these regions have since been reported to result in non-lethal and intermixed phenotypes. In the study cohort, eight individuals were found to present nonlethal outcomes despite having pathogenic glycine substitutions in the “lethal regions”. Based on our present results and other literature reports, mortality of collagen type I mutations seems to be dependent on yet unknown factors or a combination of several variables (23, 25).

In the present study, four patients were found to harbor two variants of potential causative impact located in the collagen type I genes. In each case, one of the changes was a non-glycine substitution. Performed family screening and detailed data analysis indicated that the identified non-glycine variants should be considered as not responsible for clinical manifestation of the disease. Thus, the presented cases question whether non-glycine substitutions have a deleterious impact. Moreover, it highlights difficulties that can be encountered while interpreting variants and emphasizes the importance of family testing in the context of variant prioritization. However, the possible role of non-glycine alterations as a modifying factor cannot be excluded and requires careful investigation, as similar cases have been reported (12, 17).

The phenotype spectrum observed in the Polish OI population reflects the heterogeneous nature of the disease. Although the available literature provides characteristics for each class, clinical evaluation remains subjective, as particular types present overlapping manifestations.

In our present study cohort, both skeletal and extraskeletal features were observed among patients representing all clinical types of the disease. However, prenatal presentation, vertebral fractures, triangular-shaped face, dentinogenesis imperfecta, mobility impairment, and chest and long bone deformations were significantly more common in certain forms and can be recognized as distinguishing between types. Except for vertebral fractures, the frequency of the remaining features was increasing with disease severity. In contrast, blue sclerae, hearing impairment, and osteoporosis were evenly distributed among different OI types, showing no correlation.

Comparisons with other populations are difficult, as the scope of analyzed phenotype characteristics differs between studies. However, similar trends regarding mobility and chest and long bone deformations were noted in a Ukrainian cohort (12). The most consistent observation refers to dentinogenesis imperfecta, which was noted in the Italian, Swedish, and Ukrainian populations (12, 16, 17). Statistical significance was also observed for triangular-shaped face among the Italian OI patients (16). Compared to the Polish population, vertebral fractures reported in the Swedish cohort are more frequently reported in patients with the moderate and progressively deforming OI types (17).

In several European studies, colored sclerae were observed in 33% to nearly 90% of patients assigned to type 4, which is formerly described as moderate with normal sclerae (4, 12, 16, 17). Interestingly, in our study, 78% of patients clinically attributed to this group also exhibited a colored hue, including four adults. Similar to our study, this feature was evenly reported in all three non-lethal types in the Ukrainian cohort, while blue sclera was statistically significant in the Italian and Swedish studies, being the most frequent among mild cases (12, 16, 17). This observation not only questions the initial classification of these patients but also highlights the problem of the criteria being used. Nevertheless, in those four cases, we upheld the decision, referring to other phenotypic features concordant with type 4.

Similar to earlier literature reports, some patients harboring collagen type I pathogenic variants did not exhibit any fractures or extraskeletal features (7, 15). However, the medical evaluation is based on a composite of all features present at the given time. Thus, especially in young patients, clinical presentation may change during life, with some of the diagnostic features, i.e., blue sclerae, dentinogenesis imperfecta, fractures, hearing impairment, or Wormian bones, becoming apparent at a particular age. Moreover, the natural course of the disease can be influenced by pharmacological treatment (2, 4, 28, 29).

The results of our study performed on the Polish population indicate that OI due to collagen type I gene defects presents a wide spectrum of clinical and genetic heterogeneity. As such, further studies are needed, and each one will play an important role in expanding our knowledge about the disease.

## Data availability statement

The data presented in the study are deposited in the ClinVar repository, accession numbers can be found in the **Supplementary Material Table 3**.

## Ethics statement

The studies involving humans were approved by Ethics Committee of Polish Mother’s Memorial Hospital Research Institute. The studies were conducted in accordance with the local legislation and institutional requirements. Written informed consent for participation in this study was provided by the participants’ legal guardians/next of kin.

## Author contributions

Conceptualization, KS and AG; Methodology, KS and AG; Validation, AG; Formal Analysis, KS and ŁK; Investigation, KS, IP, LR; Resources, KS, DC-S, EJ-P, IM, ŁK, AG, NW-C, MP, TC, DS, AJ, PM, MW (14th author), EP, MG, IS, MW (18th author), MR-K, AL; Data Curation, KS and DS; Writing – Original Draft Preparation, KS; Writing – Review & Editing, AG; Visualization, KS; Supervision, AG; Project Administration, KS; Funding Acquisition, KS and AG. All authors contributed to the article and approved the submitted version.
