# Supplementary material for: NGS analysis of collagen type I genes in Polish patients with Osteogenesis imperfecta: a nationwide multicenter study
Source: Front Endocrinol (Lausanne). 2023 Sep 22;14:1149982. doi: 10.3389/fendo.2023.1149982 (PMC10556695; doi:10.3389/fendo.2023.1149982)
Supplement: Supplementary file 2 [file Table_2.docx]

| **Patient** | **Variant** | **Effect** | **Status** | **Inheritance** | **Variant classification** | **OIVD** | **HGMD** | **dbSNP** | **ClinVar** | **Allele frequency** |
| --- | --- | --- | --- | --- | --- | --- | --- | --- | --- | --- |
| 7569 | **COL1A1:c.3008delC; p.Pro1003LeufsTer105** | **frameshift** | **known** | **affected mother** | **pathogenic** | **6 cases**  **OI I, OI IV** | **CD063483**  **OI I, OI IV** | **rs72653168** | **pathogenic OI I** | **not reported** |
|  | COL1A1:c.992C>T; p.Ala331Val | non-glycine substitution | novel | affected mother | likely pathogenic | not reported | not reported | rs76265813 | not reported | 0,00000882 |
| 25 | **COL1A1:c.3182G>T;**  **p.Gly1061Val** | **glycine substitution** | **novel** | **de novo** | **likely pathogenic** | **not reported** | **not reported** | **not reported** | **not reported** | **not reported** |
|  | COL1A1:c.4081G>A;  p.Glu1361Lys | non-glycine substitution | known | unaffected mother | uncertain significance | 1 case  OI IV | CM1411904  OI IV | rs141011435 | uncertain significance | 0,0000705 |
| 55 | **COL1A1:c.2155G>A;**  **p.Gly719Ser** | **glycine substitution** | **known** | **de novo** | **pathogenic** | **14 cases**  **OI III** | **CM930145**  **OI III** | **rs72651645** | **pathogenic OI I, OI III** | **not reported** |
|  | COL1A2:c.2123G>A;  p.Arg708Gln | non-glycine substitution | known | unaffected mother | likely pathogenic | 3 cases  WCT, MS, OI II | CM900074 MS? | rs72658163 | conflicting interpretation | 0,000925 |
| 103 | **COL1A1:c.2452-2A>G** | **splicing** | **known** | **affected father** | **pathogenic** | **1 case**  **OI I** | **CS063265**  **OI I** | **rs72651666** | **not reported** | **not reported** |
|  | COL1A2:c.2642A>C; p.Glu881Ala | non-glycine substitution | novel | unaffected mother | uncertain significance/ likely pathogenic | not reported | not reported | rs751201659 | uncertain significance | 0,0000615/  0,00259 |

Characteristics of double variants with potential pathogenic impact located in collagen type I genes in four patients. Variants recognized as causative for each patient are bolded. Allele frequency was based on gnomAD Exome and gnomAD GENOM databases. Abbreviations: WCT-weakness of connective tissue, MS-Marfan Syndrome.
